# A Multicentre Study: The Use of Micrografts in the Reconstruction of Full-Thickness Posttraumatic Skin Defects of the Limbs—A Whole Innovative Concept in Regenerative Surgery

**DOI:** 10.1155/2019/5043518

**Published:** 2019-12-01

**Authors:** Michele Riccio, Andrea Marchesini, Nicola Zingaretti, Sara Carella, Letizia Senesi, Maria Giuseppina Onesti, Pier Camillo Parodi, Diego Ribuffo, Luca Vaienti, Francesco De Francesco

**Affiliations:** ^1^Department of Reconstructive Surgery and Hand Surgery, University Hospital (AOU Ospedali Riuniti di Ancona), via Conca 71, 60123 Torrette di Ancona, Ancona, Italy; ^2^Department of Plastic and Reconstructive Surgery, Azienda Ospedaliero-Universitaria “Santa Maria della Misericordia”, Piazzale S. Maria della Misericordia 15, 33100 Udine, Italy; ^3^Department of Surgery “P.Valdoni”, Unit of Plastic and Reconstructive Surgery, Sapienza University of Rome, Policlinico Umberto I, Rome, Italy; ^4^Department of Plastic and Reconstructive Surgery, I.R.C.C.S. Policlinico San Donato, Università degli Studi di Milano, Via Morandi 30, 20097 San Donato Milanese, Milan, Italy

## Abstract

The skin graft is a surgical technique commonly used in the reconstructive surgery of the limbs, in order to repair skin loss, as well as to repair the donor area of the flaps and cover the dermal substitutes after engraftment. The unavoidable side effect of this technique consists of unaesthetic scars. In order to achieve the healing of posttraumatic ulcers by means of tissue regeneration and to avoid excessive scarring, a new innovative technology based on the application of autologous micrografts, obtained by Rigenera technology, was reported. This technology was able to induce tissue repair by highly viable skin micrografts of 80 micron size achieved by a mechanical disaggregation method. The specific cell population of these micrografts includes progenitor cells, which in association with the fragment of the Extracellular Matrix (ECM) and growth factors derived by patients' own tissue initiate biological processes of regeneration enhancing the wound healing process. We have used this technique in 70 cases of traumatic wounds of the lower and upper limbs, characterized by extensive loss of skin substance and soft tissue. In all cases, we have applied the Rigenera protocol using skin micrografts, achieving in 69 cases the complete healing of wounds in a period between 35 and 84 days. For each patient, the reconstructive outcome was evaluated weekly to assess the efficacy of this technique and any arising complication. A visual analogue scale (VAS) was administered to assess the amount of pain felt after the micrografts' application, whereas we evaluated the scars according to the Vancouver scale and the wound prognosis according to Wound Bed Score. We have thus been able to demonstrate that Rigenera procedure is very effective in stimulating skin regeneration, while reducing the outcome scar.

## 1. Introduction

Complex injuries of the limbs, causing crushing and loss of skin and soft tissue, occur frequently due to common injuries, both domestic and at work. The aim of surgical treatment is the morphological and functional reconstruction, allowing the recovery of the shape of the injured limbs and, at the same time, the reconstruction of its normal protective well-padded and sensitive skin which has specific properties for giving the limbs their principal functions: gait ability and grasp ability. Unfortunately, the regeneration of a specialized tissue (*i.e.*, skin) requires the restoring of the entire histological hierarchy. Usually, when the injury causes wide and deep loss of the skin and soft tissue, with bone fracture exposed, in order to achieve a functional reconstruction, using free flaps is preferable [1–3], while a useful option is covering the wound with a dermal template [4, 5]. However, in performing of all these techniques, skin grafts taken from the thighs/buttocks to directly repair the injury (or the donor site of the flap) are always essential, as well as the use of dermal substitute after its engraftment, often causing unsightly scars both at the donor and recipient site of skin grafts.

To avoid unaesthetic scars, considered an inevitable side effect of this technique, and to allow at the same time tissue regeneration of the injured site, a new innovative technology based on the application of autologous micrografts obtained by Rigenera® technology, able to induce tissue repair by highly viable skin micrografts achieved by a mechanical disaggregation method [6–8], was recently reported. A small piece of dermal/connective tissue may improve tissue repair of complex wounds [9–11] or hypertrophic scars [12]. At first, micrograft technology was applied in oral-maxillofacial surgery where micrografts derived from the human dental pulp or periosteum were used for periodontal regeneration, bone regeneration of atrophic maxilla, and alveolar socket preservation [13–16]. In the last few years, micrograft technology was applied in plastic and reconstructive surgery where micrografts derived from the cartilage were used for treatment of osteochondral lesion of the nose [17] and for enrichment of adipose tissue from human lipoaspirates [18].

Based on these considerations, the purpose of this observational study was to evaluate the efficacy of micrografts in the treatment of posttraumatic skin defects. For this reason, we have used this approach in the treatment of the posttraumatic wounds of the limbs, with excellent results in terms of clinical outcomes and demonstration of the regenerative capacity of this method by means of the tissue characterization [7].

## 2. Patients and Methods

### 2.1. Patients

From 2015 to February 2017 in four Italian Plastic and Reconstructive Units, we treated 70 patients, 38 females and 32 males with a mean age of 53 years (range 34-74 years), affected by chronic posttraumatic leg ulcer applying Rigenera protocol. All patients signed written consent to participate according to the Declaration of Helsinki. Ethics Committee approved the study (protocol N.2017-0274OR). Clinical Trial of the study is found inhttps://clinicaltrials.gov/ct2/show/NCT04030832. 24 patients suffer from bone exposure through chronic posttraumatic ulcer or surgical wound dehiscence with a mean bone exposed surface of 2 cm^2^, and 12 patients suffer from tendon exposure through chronic posttraumatic ulcer or surgical wound dehiscence. In three cases, we combined the use of Rigenera procedure with the Integra® dermal regeneration template. In one of these three patients, the Rigenera protocol was used to treat the donor site of a free flap applying the Rigenera® biocomplex over the neoderma created by Integra®. The mean time between trauma and Rigenera treatment was 7 weeks (range 2-18 weeks) (Table 1). All patients provided informed consent at the study protocol conformed to the ethical guidelines of the 1975 Declaration of Helsinki. The primary diagnosis and initial operative procedures leading to wound dehiscence are listed in Table 1. We also reported the correlated diseases affecting the patients. After an average 1-year follow-up, the evaluation of wound closure was accomplished.

### 2.2. Surgical Procedure

**Figure 1:**
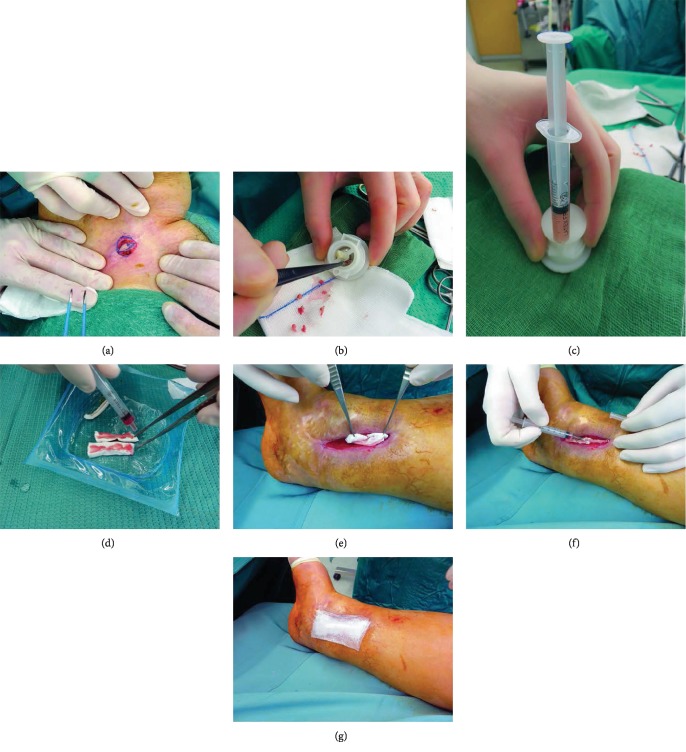
(a) Intraoperative view of skin tissue sample collection from the groin area. (b) Intraoperative view of Rigeneracons filling with 2 mm pieces obtained from the tissue sample. (c) Intraoperative view of collection of autologous micrografts obtained after the disaggregation in a sterile solution. (d) Injection of 1 ml of the micrograft solution on an equine collagen sponge. (e) Intraoperative view of Rigenera biocomplex, consisting of collagen sponge and disaggregated tissue solution, put in the wound's bed. (f) Intraoperative view of perilesional injection of 1 ml of micrograft solution. (g) Intraoperative view of tie over dressing over Rigenera biocomplex.

All cases reported in this study were treated by means of the Rigenera protocol after surgical or enzymatic ulcer's debridement and after wound infection resolution probe by culture exam. The Rigenera® technology is based on the use of the Rigenera machine and Rigeneracons (Human Brain Wave, Turin, Italy), a biological disruptor able to disaggregate small pieces of human connective tissues and select a specific cell population including progenitor cells, on the basis of cellular size. These progenitor cells, in association with the fragment of the Extracellular Matrix (ECM) and growth factors derived by starting tissue, create autologous micrografts ready for use, which can be applied on the injured area alone or in combination with different biological scaffolds, such as collagen. This protocol consists of different steps: (1) collection of a skin tissue sample of 1 cm × 1 cm from a hide donor site with respect to the recipient site (expansion ratio 1 : 10) (Figure 1(a)) (each skin sample is divided into fragments of about 2 mm^2^ each); (2) the fragments are positioned in two separated single-use capsules, below the rotating system of helical blades, resting on the filter placed on the bottom of the capsule (disaggregation of tissue for two minutes by Rigeneracons through the addition of 3 ml of sterile saline solution) (Figure 1(b)); (3) collection of 2.5 ml of autologous micrografts obtained after the disaggregation in a sterile solution (Figure 1(c)) from each capsule; (4) injection of 2.5 ml of micrograft solution on an equine collagen sponge to create a regenerative biocomplex (Figure 1(d)); (5) injection of 2.5 ml of micrograft solution into the site of injury by perilesional infiltrations and placement of the biocomplex over the ulcer taking care that the seeded surface of the sponges was in contact with the wound floor (Figures 1(e) and 1(f)); and (6) secondary medication by means of paraffin gauge tie over (Figure 1(g)).

In our patients, we collected small pieces of tissue by inguinal fold after local anaesthesia. Following the application of micrografts, all patients received for 6 days oral penicillin therapy. We performed the first dressing after 4 days without paraffin gauge removal and a second dressing after 3 days with paraffin gauge change and subjected the patients to weekly controls to evaluate the progression of wound healing. After complete healing on the treated site, moisture oil was gently applied, and after one month, the patient starts tissue massage and follow-up visit was delayed in a month's time.

### 2.3. Clinical, Pain, and Scar Evaluation

For each patient, the surface of the wounds at days 4 and 7 and every week up to complete healing has been measured and each wound was assessed for contraction. Surfaces were followed by tracing the wound edges on the computer with digital pictures. Wound contraction was measured by computer planimetry, expressed in percentage of reduction of the original wound area. At each follow-up visit, we record side effects and complications, and a visual analogue scale (VAS) was administered to assess the amount of pain felt after the micrografts' application. The pain VAS is in fact self-completed by the respondent who is asked to place a line perpendicular to the VAS line at the point representing its pain intensity. The number indicated by the respondent on the scale is recorded, and the scores range from 0 to 10, where 0 indicates pain absence and 10 severe pain [19]. Functional and aesthetic outcome was assessed using the Vancouver scale VSS [20] (height, pliability, vascularization, and pigmentation of scars) and Wound Bed Score [21] (healing time, eschar, granulation tissue, exudate, dermatitis, fibrosis, and wound bed) two months (T0) and 12 months (T1) after reepithelialisation.

### 2.4. Statistical Analysis

Application of the Shapiro-Wilk test showed data had no normal distribution; accordingly, all statistical analyses were carried out according to a nonparametric approach. To investigate the effectiveness of the Rigenera® technology, the total VSS and WBS score absolute variations between T0 and T1 were calculated, as well as the corresponding median values and their 95% Confidence Interval (95% CI). Median values were then compared using the Wilcoxon signed-rank test. Further, the percentage variation between T0 and T1 was calculated for each item of the VSS and WBS scale, and differences in each item of the two scales between T0 and T1 were investigated by means of Friedman's test.

All data were statistically analyzed using a one-way ANOVA test. The threshold for statistical significance was set at *p* values < 0.05. Repeatability is represented as a standard deviation to calculate the differences between measurements using SPSS 16.0 software (SPSS Inc., Chicago, IL, USA) for assessment.

## 3. Results

**Figure 2:**
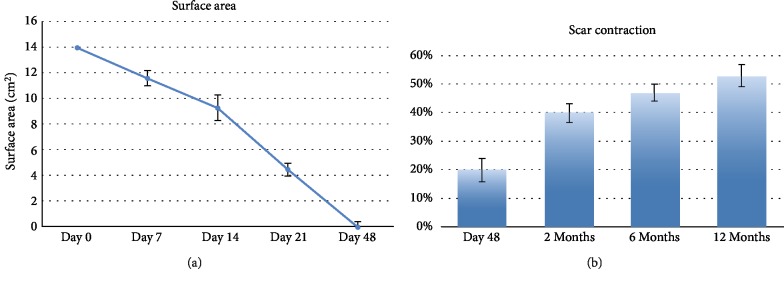
(a) Mean surfaces of the wound (cm^2^). Error bars are the standard error of the means. (b) Scar contraction. We observe contraction of all wounds at 48 days, 2 months, 6 months, and 12 months. Error bars are the standard error of the means.

**Figure 3:**
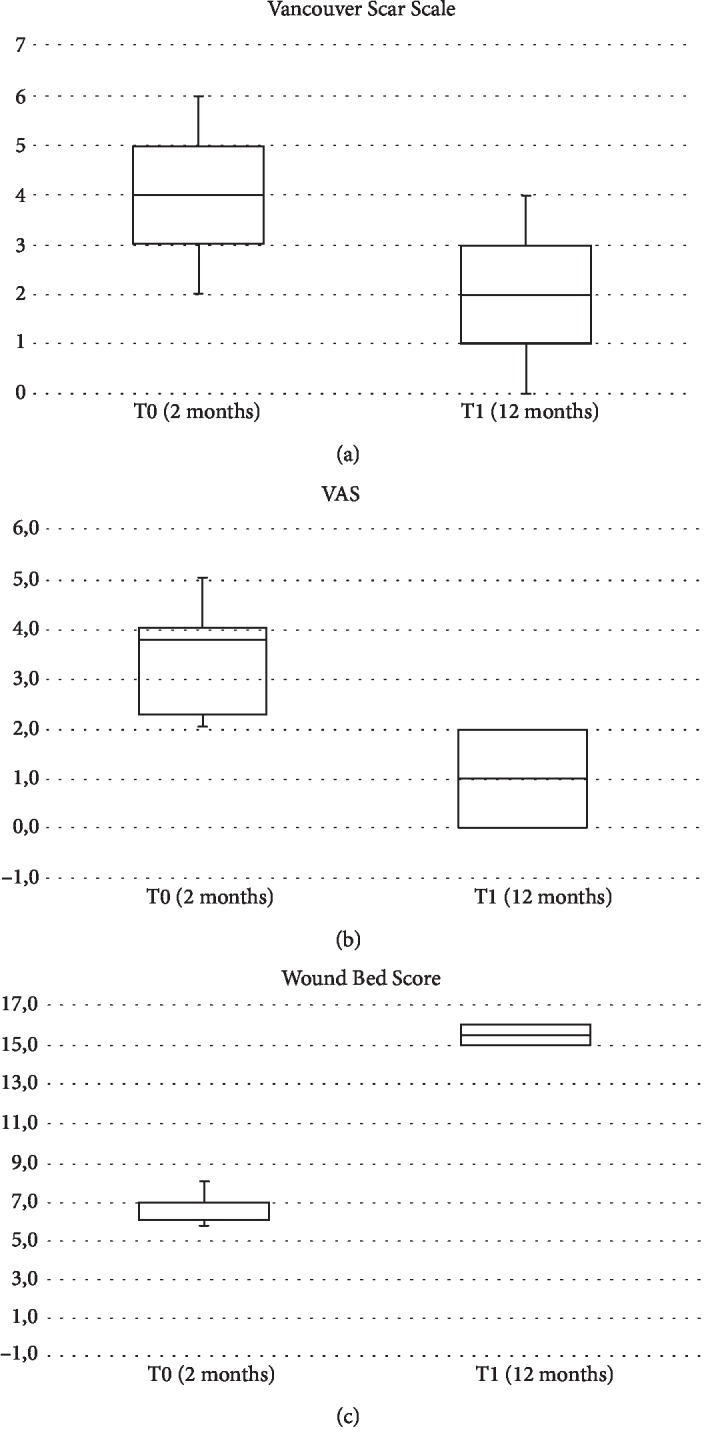
Variation of Vancouver (a), VAS (b), and WBS (c) total scores (squares, medians; bars, first and third quartiles). The reduction of the total score is significant (*p* < 0.05) in both cases.

**Figure 4:**
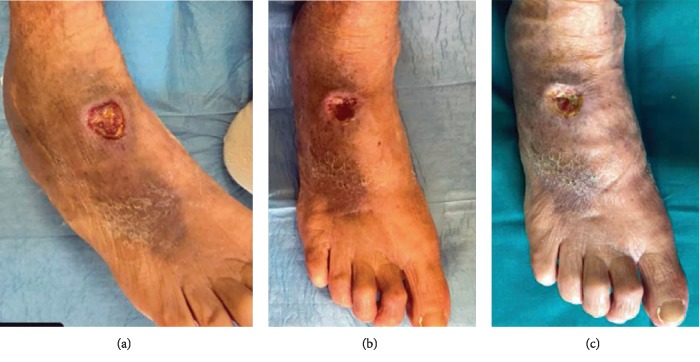
(a) Preoperative view. Dorsal foot dehiscence without bone or tendon exposure. (b) Image of the initial skin regeneration at follow-up visit 7 days after treatment. (c) Image of the initial skin regeneration at follow-up visit 30 days after treatment.

**Figure 5:**
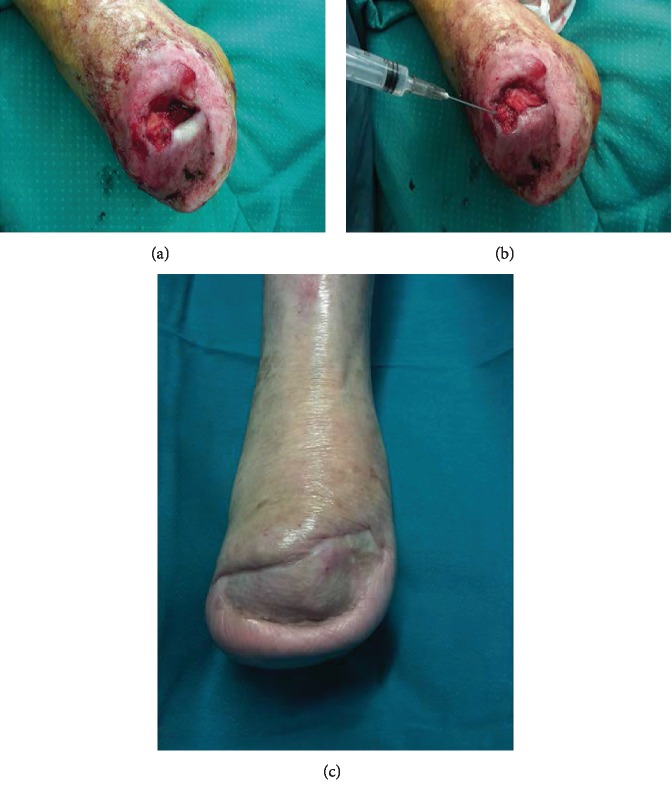
(a) Preoperative view. Foot stump dehiscence with 2 cm^2^ of bone exposure after forefoot amputation. (b) Intraoperative view of Rigenera biocomplex, consisting of collagen sponge and disaggregated tissue solution, put in the wound's bed and perilesional injection of 1 ml of micrograft solution. (c) Image of good skin regeneration at follow-up visit 6 months after complete reepithelialisation.

**Figure 6:**
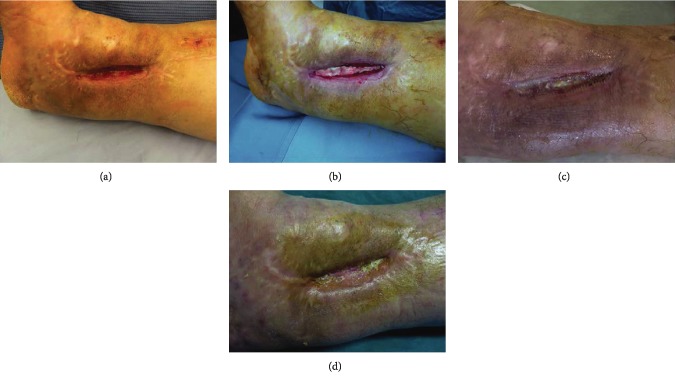
(a) Preoperative view. Ankle dehiscence without bone or tendon exposure. (b) Intraoperative view of Rigenera biocomplex, consisting of collagen sponge and disaggregated tissue solution, put in the wound's bed and perilesional injection of 1 ml of micrograft solution. (c) Image of the initial skin regeneration at follow-up visit 7 days after treatment. (d) Image of good skin regeneration at follow-up visit 6 months after complete reepithelialisation.

**Figure 7:**
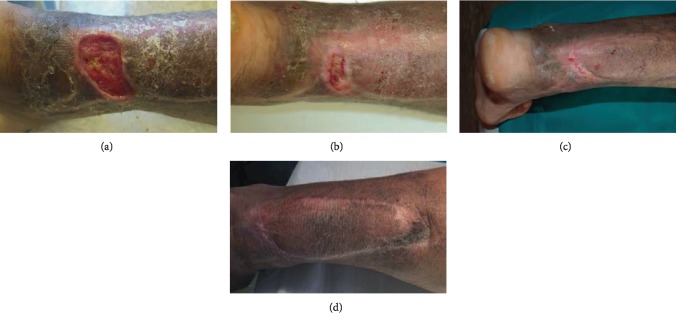
(a) Preoperative view. Achilles tendon dehiscence without bone or tendon exposure. (b) Image of the initial skin regeneration at follow-up visit 7 days after treatment. (c) Image of skin regeneration at follow-up visit 21 days after treatment. (d) Image of good skin regeneration at follow-up visit 6 months after complete reepithelialisation.

70 patients admitted to our Plastic and Reconstructive Unit were enrolled in this study. Follow-up of 90% was achieved at 1 year. Patient number 6 was removed from the study after developing Stevens-Johnson syndrome after administration of the second antibiotics. The mean age was 53 years (range 34-74 years), with 26 men (37%) and 44 women (63%). One hundred percent of wounds were located on the lower limb. Patients and wound descriptions are shown in Table 1. The origin of the soft tissue defect was open fracture in 24 patients (34%) and full-thickness skin wounds with tendon exposure in 12 patients (17%). In addition, Rigenera protocol was used in combination with Integra® dermal regeneration in 3 patients (4%). The mechanism of injury included trauma in the majority of cases (45%) and wound dehiscence (26%) and metabolic ulcer (26%) in the remaining cases. The mean time between trauma and the Rigenera treatment was 7 weeks (range 2-18 weeks). In all the other patients, we observed, on average, a complete healing of the ulcer with bone or tendon coverage in 48 days after the micrografts' application, with a range variable between 35 and 84 days. In all cases, the micrografts were applied only once; no side effect or complication was detected. In case numbers 7, 8, and 14, the micrografts were associated to Integra®. In cases 7 and 8, it was employed as secondary dressing; in these patients, the complete healing was achieved without a secondary surgical procedure of skin grafting. In case 14, the micrografts were put over the dermal substitute 30 days after its engraftments in order to achieve complete reepithelialisation without the secondary procedure of skin graft. In these three cases, wound healing was obtained after, respectively, 35, 42, and 35 days. At day 0, surfaces of every wound were about 14 cm^2^ (range 7-28 cm^2^). At day 7, mean surfaces were 11.6 cm^2^ (standard error of the mean was ±2.3 cm^2^). At day 14, mean surfaces were 9.3 cm^2^ (standard error of the mean was ±1.6 cm^2^). At day 21, mean surfaces were 4.5 cm^2^ (standard error of the mean was ±2.1 cm^2^). At day 48, mean surfaces were 0 cm^2^ (standard error of the mean was ±1.3 cm^2^) (Figure 2(a)). Moreover, we observed contraction of all wounds after the complete closure. Wound contraction percentage was significantly different from 20% at day 48 (standard error of the mean was 4%, *p* < 0.001) to 40% at day 60 (standard error of the mean was 3.4%, *p* < 0.001) (Figure 2(b)). The mean preoperative VAS score was 6 (ranging from 4 to 9); meanwhile, the mean VAS score at the first follow-up visit was 3 (ranging from 2 to 5) (Table 2(a)). The Vancouver Scar Scale (VSS) was used to evaluate functional and aesthetic characteristics of lesions after 2-month (T0) and 12-month (T1) follow-up as shown in Table 2(b). There was no statistical difference in sex, age, underlying disease, and size of the defect. The VSS mean value was 2 (ranging from 0 to 4). The VSS score showed a significant reduction at the T1 follow-up control visit (*p* < 0.05). The WBS mean value was 15.4. The WBS score showed a significant reduction at the T1 follow-up control visit (*p* < 0.05) (Table 2(c)). Results concerning the single items of VSS and WBS scale are summarized in Table 2. The scores showed a significant reduction at the T1 (12 months) follow-up control visit (*p* < 0.05): the median Vancouver total score decreased by 12 units (-85.7%) and the median VAS score decreased by 9 units (-90%) while the median WBS increased by 158% (Figures 3(a)–3(c)). The VSS and VAS scores decreased after the 12-month follow-up, the greatest reduction being observed in height, pliability, and pigmentation. All scars were supple, with a normal height and a normal pigmentation. The only difference between T0 and T1 was about the vascularization and height. The WBS score increased after the 12-month follow-up, the greatest increase being observed in healing edges, exudate amount, and depth/granulation tissue. We have used this technique in 70 cases of chronic wounds of the lower limbs, characterized by extensive loss of skin substance and soft tissue. In particular, a more fast and complete reepithelialisation with respect to the other advanced dressings that were previously utilized in the treatment of the traumatic wounds of the limbs has been observed, with excellent results (Figures 4–7).

## 4. Discussion

The skin grafts are a surgical technique commonly used in the reconstructive surgery of the limbs, in order to directly repair skin loss, as well as to repair the donor area of the flaps and cover the dermal substitutes after engraftment. The inevitable side effect of this technique consists of unaesthetic scars. The autologous tissue grafts produce very evident scars and are unable to stimulate tissue regeneration, because the interruption of blood circulation leads to an extensive inner cell death. This issue is related to the prevalence of scarring on skin regeneration in the management of traumatic wounds and is even more evident in patients affected by chronic diseases who are highly exposed to the risk of delayed healing of the injured tissue leading to a pathological inflammatory state and chronic wounds [22]. Researchers are trying to find approaches able to reduce scarring after wound healing, stimulating at the same time tissue regeneration, by means of tissue engineering methods. Tissue engineering aims to regenerate tissues through the combined use of biomaterials and biologic mediators such as stem cells and growth factors in order to provide new tools for regenerative medicine [23, 24]. The repair and regeneration of human tissues is particularly difficult in skeletal reconstruction of large bone defects caused by trauma, infection, or skeletal abnormalities, and given that regenerative ability of bone declines with increasing age, the dramatic rise in the ageing population worldwide determined an increasing need for innovative approaches [25]. Many approaches can be used when the normal process of tissue regeneration is impaired or simply insufficient, such as grafting which includes autografts, allografts, xenografts, and biomaterial substitutes [26]. For the safety of grafting procedure, the autologous grafts are preferable to those homologous or heterologous grafts, and although it prevents immunoreactions and infections, this approach is limited due to additional surgical procedure on the donor site and discomfort for the patient. Furthermore, about the autologous grafts, the cell viability dramatically decreases after collection from the donor site for vessel interruption by surgery with reduced feeding for the cells. The Rigenera® technology represents a new approach for human injured tissue regeneration, and when using this technology, the patient is the donor and acceptor of calibrated and highly viable micrografts containing progenitor cells positive for mesenchymal stem cell markers able to induce tissue repair [7, 11]. In vitro data on their characterization have reported that micrografts display a mesenchymal phenotype and are positive for typical markers such as CD73, CD90, CD105, and CD117 and have showed their capability to differentiate in osteocytes, chondrocytes, or adipocytes in appropriate experimental conditions [13, 31]. Furthermore, the clinical efficacy of these micrografts has been demonstrated both in the healing of postoperative and posttraumatic wounds [9–11, 27] and pathological scars [12]. In particular, a more fast and complete reepithelialisation with respect to the other advanced dressings that were previously utilized in the treatment of the traumatic wounds of the limbs has been observed, with excellent results in terms of clinical efficacy and demonstration of the regenerative capacity of this method [7, 8]. The micrografting concept was conceived by Cicero Parker Meek and was based on the evidence that skin grafts expanded many times were able to heal a wound faster than the original-sized grafts [28]. Therefore, the best way to achieve a good grafting performance is by increasing the superficial area of the graft leading to a faster cellular migration onto the wound and reducing cellular death of the graft itself. Since then, the micrograft concept was applied to many procedures [29, 30].

Rigenera technology is different from any of them because it is able to generate a suspension of micron-sized grafts (micrografts) which are applied with the help of a syringe. The average size of the Rigenera-obtained micrografts is 80 *μ*m, extremely smaller than any other technology available on the market or ever described in literature. Lastly, while performing the disaggregation, this technology allows for a collection of only the smaller cells which also express the mesenchymal stem cell markers, described as stem cell-like or progenitor cells, which are accounted for a strong regenerative effect [13, 31]. We have used this technology in 70 cases of chronic wounds of the lower limbs, characterized by extensive loss of skin substance and soft tissue. Some of these patients were affected by severe chronic diseases with poor vascularization of the skin. In all cases, we have applied the Rigenera protocol using skin micrografts, achieving in 69 cases the complete healing of wounds in a period between 35 and 84 days. Wound healing was characterized by complete reepithelialisation with clinical and histological evidence of a repair that occurred through a process of tissue regeneration and low presence of scar at the injured area [7, 8]. The regenerative efficacy of micrografts could arise from the presence of small particle-sized autografts characterized by a large grafting surface, leading to an optimal cellular viability and integrity, for minor nutritional needs of the cells. Particularly, the mechanical disaggregation of small pieces of human connective tissues produces a suspension of autologous micrografts of 80 *μ*m size, ready for use, which can be applied on the injured area alone or in combination with different biological scaffolds, such as collagen and hyaluronic acid. The specific cell population of these micrografts includes progenitor cells, which in association with the fragment of the Extracellular Matrix (ECM) and growth factors derived by starting tissue initiate biological processes of cell proliferation and differentiation enhancing the wound healing process. This is the “micrograft theory” that may explain the excellent reconstructive outcomes in the treatment of the injured limbs applying autologous micrografts obtained by the Rigenera® technology.

## 5. Conclusions

The autologous micrografts obtained by the Rigenera® technology are an innovative protocol that introduces a whole new concept in regenerative surgery, allowing to repair severe traumatic wounds of the limbs by complete reepithelialisation. The impressive clinical outcomes combined with the laboratory tests on the tissue characterization demonstrate that this technology is really able to stimulate skin regeneration and probably it is the only one available today able to ensure the healing of traumatic injuries through a real regenerative procedure. In addition, the minimum amount of skin required to produce micrografts with the Rigeneracons in the repair of wide traumatic wounds prevents scarring usually produced by traditional techniques at the donor site.

Definitely, this innovative technology is not just a new technique but a whole new concept in regenerative surgery.

## Figures and Tables

**Table 1:** Patients and wound description.

	*n* (%)	Mean value	Minimum-maximum
**Patients**			
Total	70 (100%)		
Sex			
Male	32 (23%)		
Female	38 (27%)		
Age		53 years	34-74 years
Underlying disease			
None	49 (70%)		
Diabetes type II	15 (22%)		
Arteriopathy	6 (8%)		
**Wound description**			
Wound location			
Thigh	6 (8%)		
Leg	30 (43%)		
Ankle	19 (28%)		
Foot	15 (21%)		
Type of injury			
Wound dehiscence	18 (26%)		
Posttraumatic ulcer	32 (45%)		
Metabolic ulcer	18 (26%)		
Burn	2 (3%)		
Surface area		14 cm^2^	7-28 cm^2^
Exposed structures			
None	34 (49%)		
Tendon	12 (17%)		
Bone	24 (34%)		
**Rigenera procedure**			
Infection			
Before procedure	8 (11%)		
After procedure	0 (0%)		
Rigenera treatment delay after trauma		7 weeks	2-18 weeks
Antibiotics postoperative	70 (100%)	6 days	6 days
Time of complete healing		48 days	35-84 days
None (S-J syndrome)	1 (1%)		
Complication/note			
None	66 (95%)		
Stevens-Johnson syndrome	1 (1%)		
Associate use (Rigenera+Integra®)	3 (4%)		

**Table 2:** 

	Pretreatment	T0 2-month follow-up	T1 12-month follow-up	*p* value
(a) **Results of VAS score**				
VAS score	6 (9-4)	3,4 (5-2)	1 (2-0)	*p* < 0.05
(b) **Results of Vancouver Scar Scale**				
Vascularity		2	0	*p* = 0.003
Pigmentation		0	0	*p* = 0.5
Pliability		1	1	*p* = 0.5
Height		2	1	*p* = 0.016
Total score		4,1 (6-2)	2,03 (4-0)	*p* < 0.05
(c) **Results of Wound Bed Score scale**				
Healing edges	0	1	2	*p* < 0.05
Black eschar	1	2	2	*p* = 0.5
Greatest wound	0	1	2	*p* = 0.5
Depth/granulation tissue	0	0	2	*p* < 0.05
Exudate amount	0	2	2	*p* < 0.05
Edema	1	1	2	*p* = 0.5
Periwound dermatitis	0	0	1	*p* = 0.5
Periwound callus/fibrosis	0	0	1	*p* = 0.5
Pink wound bed	0	0	1	*p* = 0.5
Total score	3	7	15	*p* < 0.05

## Data Availability

The data used to support the findings of this study are included within the article.
